# DHX9 regulates production of hepatitis B virus-derived circular RNA and viral protein levels

**DOI:** 10.18632/oncotarget.25104

**Published:** 2018-04-20

**Authors:** Kazuma Sekiba, Motoyuki Otsuka, Motoko Ohno, Takahiro Kishikawa, Mari Yamagami, Tatsunori Suzuki, Rei Ishibashi, Takahiro Seimiya, Eri Tanaka, Kazuhiko Koike

**Affiliations:** ^1^ Department of Gastroenterology, Graduate School of Medicine, The University of Tokyo, Tokyo 113-8655, Japan

**Keywords:** HBV, cccDNA, minicircle, primary hepatocytes, digital PCR, Immunology

## Abstract

Hepatitis B virus (HBV) infection, which is a major health concern worldwide, can lead to liver cirrhosis and hepatocellular carcinoma. Although current nucleos(t)ide analogs efficiently inhibit viral reverse transcription and viral DNA load clinically, episomal viral covalently closed circular DNA (cccDNA) minichromosomes and transcripts from cccDNA continue to be expressed over the long term. We hypothesized that, under these conditions, viral transcripts may have biological functions involved in pathogenesis. Here, we show that the host protein DExH-box helicase 9 (DXH9) is associated with viral RNAs. We also show that viral-derived circular RNA is produced during HBV replication, and the amount is increased by knockdown of the DHX9 protein, which, in turn, results in decreased viral protein levels but does not affect the levels of HBV DNA. These phenomena were observed in the HBV-producing cell culture model and HBV mini-circle model mimicking HBV cccDNA, as well as in human primary hepatocytes infected with HBV. Based on these results, we conclude that, in HBV infection, the RNA binding factor DHX9 is a novel regulator of viral circular RNA and viral protein levels.

## INTRODUCTION

Despite the availability of effective vaccines, hepatitis B virus (HBV) infection remains a major global health problem. Approximately 2 billion people are infected with HBV, and over 350 million patients are chronic HBV carriers [1]. Chronic hepatitis B is a risk for liver cirrhosis and hepatocellular carcinoma, and more than 700,000 people die from HBV-associated diseases each year [2].

HBV is a DNA virus with a 3.2-kb partially double-stranded relaxed circular DNA (rcDNA) as its genome. Covalently closed circular HBV DNA (cccDNA) is formed by conversion from rcDNA following HBV infection. HBV cccDNA exists persistently in the hepatocyte nucleus in an episomal state, where it acts as a viral transcription template. Host RNA polymerase II transcribes HBV genes to produce five viral RNAs: a transcript that encodes precore, a transcript that encodes core and pregenomic RNA (pgRNA), a preS1 transcript that encodes the large S surface protein, a transcript that encodes preS2 and S protein (middle S and small S proteins), and the X gene mRNA [3]. The three surface proteins collectively comprise HBsAg. All transcripts are encoded in overlapping reading frames and have the same 3’ end with polyadenylation signals. Although these RNAs were initially considered to be only templates for viral protein translation and intermediates for the synthesis of viral DNA by reverse transcription for replication, it has recently been discovered that these RNAs may also have crucial pathological functions [4–6].

It has been recently revealed that RNAs, especially non-coding RNAs, have diverse biological functions [7]. Although nucleos(t)ide viral replication inhibitors efficiently inhibit viral reverse transcription and viral DNA load [8, 9], episomal viral cccDNA, as well as transcripts and proteins from cccDNA, continue to be expressed. In this context, although HBV RNAs have been considered to be simply the template for protein synthesis and viral DNA replication, they may also exhibit additional biological functions involved in pathogenesis (e.g., acting as a decoy for specific microRNAs (miRNAs)), as we and others have previously reported [4, 10]. Moreover, when HBV-DNA levels are quite low under the treatment of nucleos(t)ide analogs, HBV protein or transcript levels are associated with the risk of hepatocellular carcinoma [11].

In this study, to discover unknown biological functions of HBV RNAs, host proteins that can associate with viral RNAs were comprehensively identified using RNA immunoprecipitation followed by proteomic approaches. We found that circular RNA is produced from HBV RNA and that the host RNA-binding protein DExH-box helicase 9 (DXH9) is involved in its production. Knockdown of DXH9 leads to increased production of this viral circular RNA but to decreased viral surface protein levels. These results represent novel findings regarding HBV RNAs and the regulation of viral protein levels.

## RESULTS

### Host proteins interacting with HBV mRNAs

**Figure 1:**
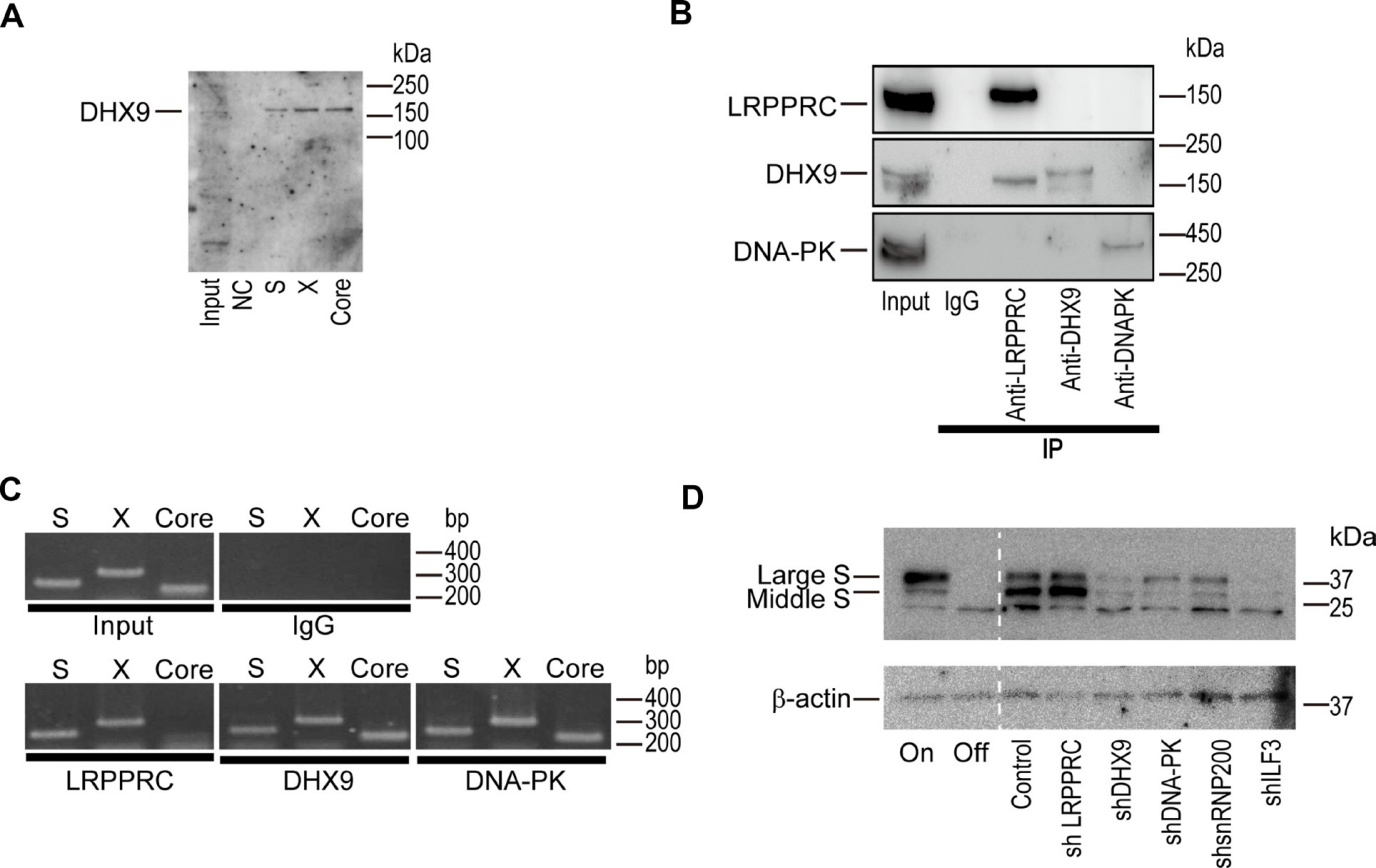
DExH-box helicase 9 (DHX9) knockdown downregulates viral protein levels (**A**) DHX9 co-precipitated with HBV RNAs. RNAs corresponding to each region (S, X, and Core) were mixed with HepG2 cellular lysates *in vitro* and precipitated. Co-precipitated protein was blotted with anti-DHX9. Five percent of the cell lysates was used as an input. NC: negative control (no RNA). A representative image of two independent experiments is shown. (**B**) HepAD38 cells with hepatitis B virus (HBV) expression were used for the immunoprecipitation of the indicated proteins. Western blotting was used to confirm immunoprecipitation of the targeted proteins. Five percent of the cell lysates was used as an input. Normal rabbit IgG was used as a negative control for the immunoprecipitation. A representative image of two independent experiments is shown. (**C**) RNA was extracted from the precipitants by the indicated proteins and was subjected to reverse transcription-polymerase chain reaction (RT-PCR) to determine if the indicated RNA regions (S, X, Core) were included. A representative image of two independent experiments is shown. (**D**) HepAD38 cells with stably expressing short hairpin RNAs (shRNAs) against the indicated proteins were used for determining the expression levels of the HBV S protein. Before the assay, doxycycline (1.0 μg/ml) was added to the cells for 3 days to shut off the newly synthesized HBV transcripts. HepAD38 cells continuously treated with (Off) or without (On) doxycycline were included as controls. A representative image of two independent experiments is shown.

To comprehensively identify host proteins that interact with HBV-derived RNAs, we performed an RNA precipitation assay after mixing cell lysates with biotinylated HBV-RNAs including the S, X, and core regions. After precipitation, proteins were dissolved by sodium dodecyl sulfate-polyacrylamide gel electrophoresis (SDS-PAGE), and possible interacting protein bands were excised for identification by mass spectrometry (Supplementary Figure 1A). Among the 15 candidate proteins identified, we confirmed the RNA-protein binding by western blotting for six proteins, including DHX9 (Supplementary Figure 1B and Figure 1A). Conversely, after immunoprecipitation of candidate proteins from HepAD38 cells in which HBV replicates [12], the RNAs interacting with these proteins were extracted and subjected to polymerase chain reaction (PCR) to determine if the interacting RNAs included HBV-derived RNAs. Under the conditions in which candidate proteins were precipitated, HBV-RNAs, including most open reading frame regions, were confirmed to interact with the identified proteins (Supplementary Figure 1C, 1D and Figure 1B, 1C). To determine whether these proteins were involved in HBV replication, we established stably short hairpin RNA (shRNA)-expressing HepAD38 cells in which each candidate protein was knocked down. Knockdown of DHX9, DNA-dependent protein kinase (DNA-PK), small nuclear ribonucleoprotein 200 kDa (snRNP200), or interleukin enhancer binding factor 3 (ILF3) (Supplementary Figure 2A) led to a decrease in the protein levels of HBV large and middle S (Figure 1D), suggesting that these RNA-interacting proteins work as enhancers of HBV replication or viral protein expression levels.

### Circular RNA derived from HBV

**Figure 2:**
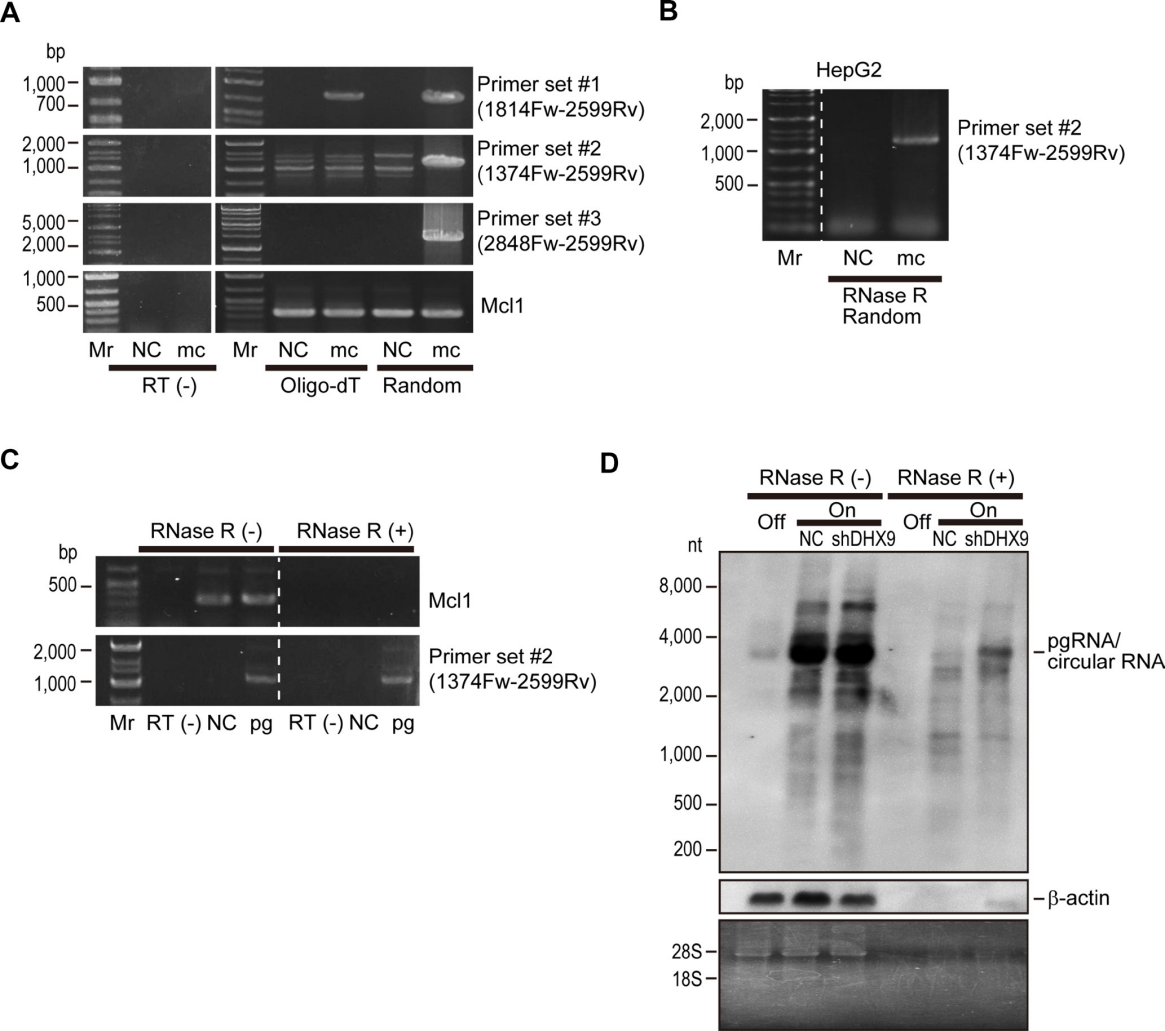
Viral circular RNA is produced during HBV replication (**A**) HepG2 cells were transfected with HBV minicircle construct (mc). Three days after transfection, RNAs were extracted and subjected to complementary DNA (cDNA) synthesis by reverse transcription using oligo-dT (Oligo-dT) or random (Random) primers as indicated. Cells without transfection were used as negative controls (NC). PCR was performed using the indicated primer sets. Primer set #1 was used to amplify the core region, and set #2 and set #3 were outward facing primers, which would theoretically result in no amplification. Mcl1 amplification was used as an internal control. RNAs that were not reverse transcribed (RT(−)) were included as negative controls for the PCR to confirm no DNA contamination. Mr, DNA marker. A representative image of two independent experiments is shown. (**B**) RNAs extracted from HepG2 cells transfected with HBV minicircle construct (mc) for 3 days were treated with RNase R and subjected to RT-PCR using random primers and primer set #2. Untransfected cells were used as a negative control (NC). (**C**) HepG2 cells were transfected with HBV pgRNA expressing plasmid (pg). Two days after transfection, extracted RNA was treated with or without RNase R and reverse transcribed using random primers. PCR was performed using primer set #2 and primers for Mcl1 gene amplification as a control. RNAs that were not reverse transcribed (RT(−)) were used as negative controls. Mr, DNA marker. A representative image of two independent experiments is shown. (**D**) DHX9 knockdown leads to increased viral circular RNA production. HepAD38 cells with HBV shut off (Off), HBV continuous expression (On), and HBV continuous expression with shDHX9 expression (On + shDHX9) were subjected to northern blotting with or without RNase R treatment. Probes against HBV RNA primarily spanning the core region and β-actin were used. Gel staining with ethidium bromide is shown to confirm RNA integrity (bands of 28S and 18S ribosomal RNA) and the effects of RNase treatment (disappearance of ribosomal RNA bands). A representative image of two independent experiments is shown.

To determine whether knockdown of the identified proteins affected HBV replication or HBV-DNA levels, we performed southern blotting to detect HBV-derived DNA levels including open circular DNA, double-stranded linear DNA, and cccDNA levels in HepAD38 cells with knockdown of each protein. Southern blotting revealed that HBV-derived DNA levels were not significantly changed by knockdown of any of the identified proteins (Supplementary Figure 2). Thus, we examined if HBV-RNA levels were altered by knockdown of the candidate proteins. To this end, we examined HBV RNA levels from an HBV minicircle construct, which mimics HBV cccDNA. We prepared three primer sets for PCR amplification of HBV RNAs (primer set #1: 1814 Fw and 2599 Rv; primer set #2: 1374 Fw and 2599 Rv; and primer set #3: 2848 Fw and 2599 Rv; the numbers reflect the positions of the HBV genome). Whereas primer set #1 amplified a core region of the pregenomic RNA, primer sets #2 and #3 were assumed to act as negative controls because the primers are theoretically not able to amplify any amplicons from HBV linear mRNAs (Supplementary Figure 3). However, unexpectedly, we detected amplicons derived from minicircle HBV when primer sets #2 and #3 were used, although this occurred only when reverse transcription was performed using random primers and not oligo-dT primers (Figure 2A). We confirmed that these amplicons were not derived from contaminated minicircle HBV-DNA, because amplicons could not be detected using RNAs without reverse transcription (Figure 2A). These results led us to hypothesize that some circular RNAs are produced from HBV-RNA. To confirm this, we treated RNAs from HBV minicircle-transfected HepG2 cells with RNase R, an exoribonuclease, which specifically digests linear RNAs but not circular RNAs, before reverse transcription-PCR (RT-PCR). Following RNase R treatment, using primer set #2, we could detect an amplicon from RNAs transfected with the HBV minicircle, suggesting that circular RNA, which remains intact following RNase R treatment, was produced from the HBV minicircle (Figure 2B).

To further confirm the existence of circular RNA derived from HBV-RNA, we next examined RNAs derived from cells transfected with HBV pgRNA-expressing plasmids. Whereas RNase R treatment clearly digested host linear Mcl1 mRNA, primer set #2 could amplify the amplicon derived from HBV RNA even after RNase R treatment (Figure 2C). These results suggested that HBV pgRNA could produce circular RNA.

Among the proteins identified as interacting with HBV-RNAs, DHX9 was recently reported to be involved in the production of circular RNAs. In particular, the loss of DHX9 leads to an increase in circular RNAs [13]. We decided to focus on DHX9 and examined the changes in HBV circular RNA levels by northern blotting in shDHX9-expressing HepAD38 cells with and without RNase R treatment. The probe for northern blotting was generated by amplification of the core region of HBV RNA and should theoretically detect only HBV pgRNA. In fact, dense bands at around 3.2 kb (the length of the HBV genome) were observed in the absence of RNase R treatment irrespective of DHX9 knockdown. These bands should include pgRNA and possible circular RNAs. Indeed, following RNase R treatment, the bands became weaker, likely because pgRNAs were digested, similar to the β-actin mRNA. However, some RNA remained, which was more apparent in the case of DHX9 knockdown (Figure 2D). These results suggested that viral circular RNA, with a size similar to that of pgRNA, is produced from HBV-RNA and that knockdown of DHX9 increases the levels of this circular RNA.

### DHX9 interacts with HBV circular RNA

**Figure 3:**
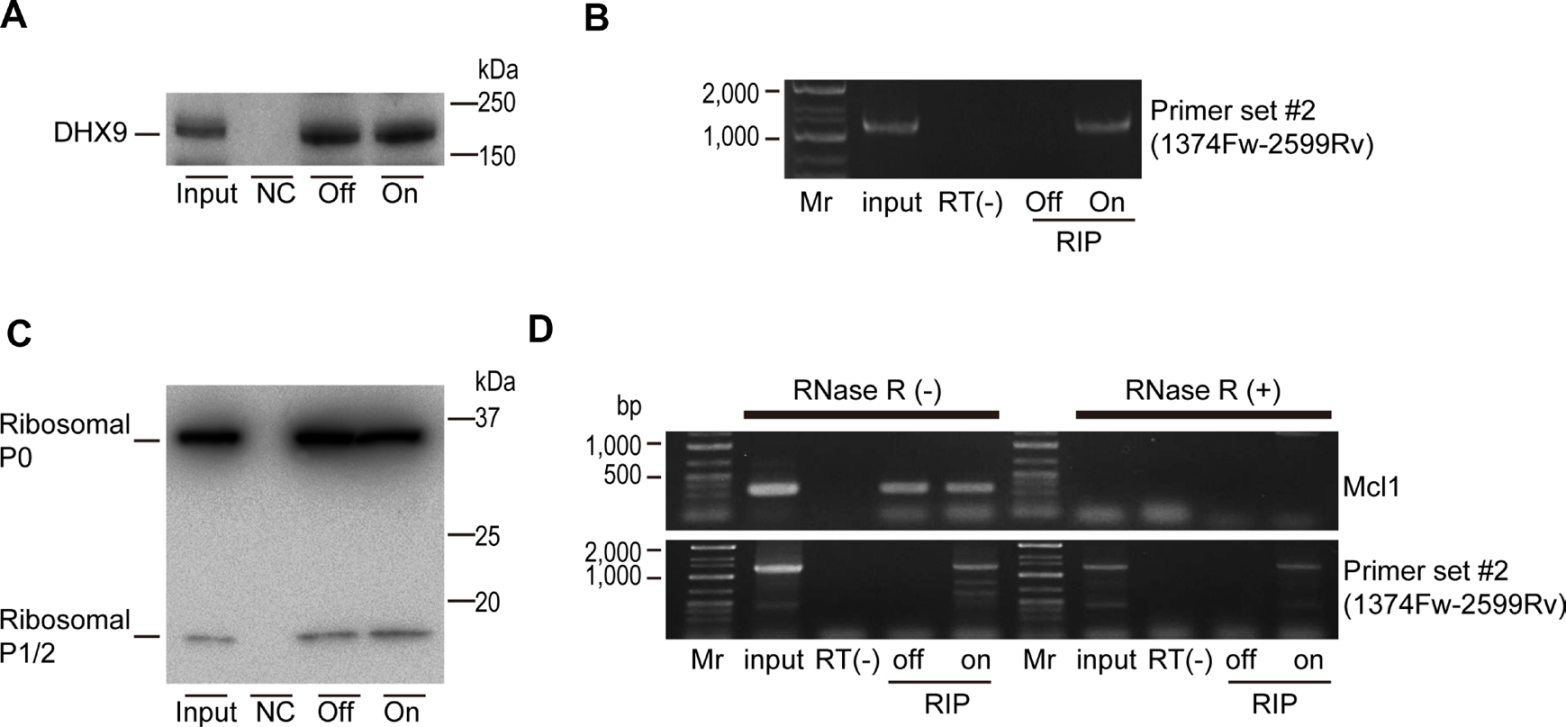
DHX9 and ribosomal proteins are associated with viral circular RNA (**A**) HepAD38 cells with or without HBV expression (On or Off) were used for DHX9 precipitation. Normal rabbit IgG was used for the negative control (NC). DHX9 precipitation was confirmed by western blotting. A representative image of two independent experiments is shown. (**B**) RNAs were extracted from the immunoprecipitations and reverse transcribed using random primers. PCR was performed using primer set #2. RNAs that were not reverse transcribed were included as negative controls (RT(−)). Mr, DNA marker. A representative image of two independent experiments is shown. (**C**) Ribosomal P0/P1/P2 protein (Ribo0-2) was immunoprecipitated as described in (a). Five percent of the cell lysates was used as an input. A representative image of two independent experiments is shown. (**D**) Extracted RNAs from the immunoprecipitations (RIP) were treated with or without RNase R, followed by reverse transcription using random primers. PCR was performed using primer set #2 and MCl1 gene primers as a control. RNA extracted directly from cells was used as an input. RNAs that were not reverse transcribed were included as negative controls (RT(−)). Mr, DNA marker. A representative image of two independent experiments is shown.

As noted above, DHX9 was identified as an HBV-RNA-interacting protein. To examine whether DHX9 also interacted with HBV circular RNA, we performed an RNA-immunoprecipitation (RIP) assay by pull-down of the DHX9 protein in HepAD38 cells with or without HBV expression. Precipitation of a similar amount of DHX9 protein was confirmed under both conditions (Figure 3A). Because the amplicon generated using primer set #2, assumed to be a result of circular RNA, could only be amplified from the precipitated RNA when HBV was expressed, it was suggested that HBV-derived circular RNA exists in the co-precipitated RNA with DHX9 (Figure 3B). Thus, the DHX9 protein appears to interact with HBV circular RNA.

To determine if the HBV circular RNAs interacted with the host factors involved in translation, we next performed an RIP assay using an antibody against the ribosomal P0/P1/P2 protein. Because the ribosomal proteins P0, P1, and P2 exist as a pentameric complex, P0(P1)2(P2)2, on the large subunits of eukaryotic ribosomes, this complex plays a crucial role in the recruitment of translation factors to the ribosome [14]. The RIP assay was performed using HepAD38 cells with and without HBV expression (Figure 3C). As expected, host gene Mcl1 linear mRNA could be detected in the immunoprecipitation, which was decreased following RNase R treatment (Figure 3D). However, the amplicon obtained using primer set #2 before RNase R treatment could be detected only when HBV was expressed, and it was also detected after RNase R treatment (Figure 3D). These results suggested that HBV circular RNA interacts with ribosomal proteins. However, because knockdown of the DHX9 protein led to a decrease in viral proteins (Figure 1D), this result may indicate that the association between ribosomal proteins and circular RNA does not always indicate the occurrence of translation from these circular RNAs.

### HBV infection produces circular RNAs

**Figure 4:**
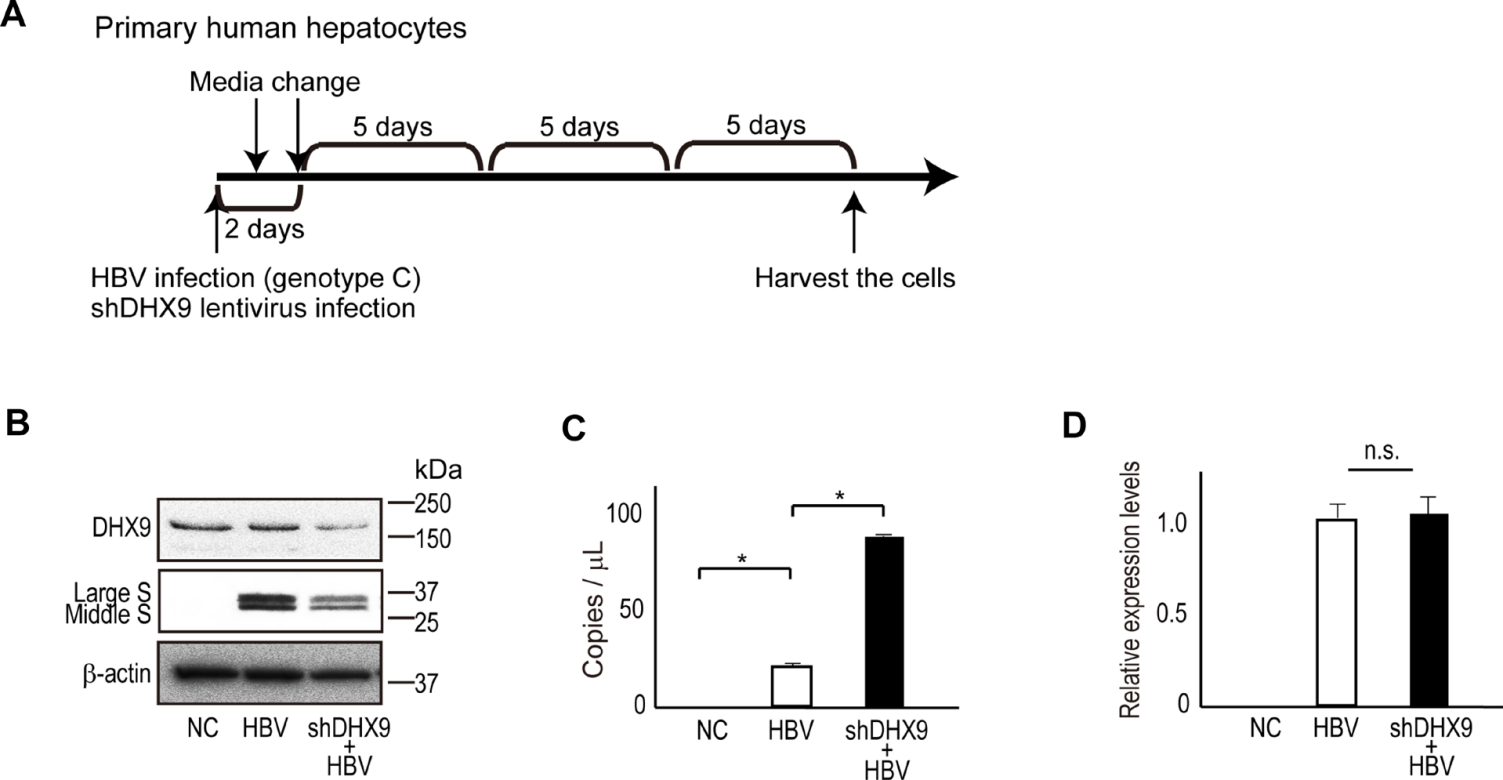
DHX9 regulates viral circular RNA production and viral protein levels (**A**) A scheme for HBV infection in human primary hepatocytes. At HBV infection, lentivirus-expressing shDHX9 was also applied simultaneously to knock down DHX9. Cells were harvested at 17 days after infection. (**B**) Cell lysates were subjected to western blotting to determine DHX9 and HBV surface protein levels in DHX9-knockdown cells. (**C**) RNAs extracted from the HBV-infected human primary hepatocytes were treated with DNase and subsequently with RNase R, followed by reverse transcription using random primers. cDNAs were subjected to droplet digital PCR using Taqman primers for the absolute quantification of HBV circular DNA. The measurement was performed in triplicate. Data represent the mean ± standard error (SE) of the absolute copy numbers from two independent experiments. ^*^p < 0.05. (**D**) Real-time PCR was performed to measure pgRNA levels (including circular RNA) using the total RNAs from HBV-infected human primary hepatocytes. Following DNase treatment, reverse transcription was performed using random primers, and real-time PCR amplifying the core region was performed in triplicate. The data were normalized with β-actin. Data represent the mean ± SE of two independent experiments. n.s., no significant differences.

Finally, to determine if natural HBV infection also produces HBV circular RNA, we examined an *in vitro* HBV replication model using primary human hepatocytes. On day 1, primary hepatocytes were infected with HBV genotype C with or without shDHX9-expressing lentiviruses. The media was changed the following day as well as 2 days after infection. After that, the media was changed every 5 days; at 17 days after infection, cells were harvested for further analyses (Figure 4A). Consistent with the results discussed above, the protein levels of HBV large S and middle S were significantly decreased in DHX9-knockdown cells (Figure 4B). To determine if natural HBV replication produced circular RNAs, we examined RNAs by RT-PCR following RNase R treatment. Because the amount of HBV circular RNA, if any, was predicted to be low, we applied the droplet-digital PCR (ddPCR) method for the exact quantification of minute samples. Primers assumed to detect HBV cccDNA were used for the detection of HBV circular RNA following DNase treatment and reverse transcription. We observed that HBV circular RNAs were indeed produced during HBV replication, and knockdown of the DHX9 protein led to an approximately three-fold increase in the number of HBV circular RNAs (24 vs. 75 copies in 1 μl complementary DNA [cDNA]) (Figure 4C). Because 2.5% of total RNA from HBV-infected cells in one well of a 24-well plate were theoretically subjected to the ddPCR assay, 960 copies of circular RNA were assumed to exist in approximately 3 × 10^5^ cells in this scale and system. Because the levels of pgRNA, including circular RNA, determined by quantitative RT-PCR using primers corresponding to the core sequences were comparable between wild-type and DHX9-knockdown cells (Figure 4D), the levels of the RNA template for viral protein translation were likely reduced in DHX9-knockdown cells because of the existence of more circular RNA. These results suggest that HBV produces circular RNA and that the knockdown of DHX9 leads to an increase in viral circular RNA, which, in turn, decreases the levels of the RNA templates for viral protein translation and viral protein expression levels.

## DISCUSSION

Herein, we showed that circular RNA is produced during HBV replication, and the levels are regulated by the RNA-binding protein DHX9, which, in turn, may be a crucial factor in the regulation of viral protein levels. To the best of our knowledge, these are novel findings in the HBV research field.

Circular RNA is a type of non-coding RNA. Because circular RNAs do not have 5′ and 3′ ends, they are generally more stable than linear coding or non-coding messages [15]. However, because it has been difficult to study circular RNAs with traditional methods of RNA analysis, information about these species has remained limited [16]. Yet, some specific pathogens, such as hepatitis delta virus and some plant viroids, have been known to produce circular RNAs [17, 18]. In this study, we identified circular RNA production from HBV by two methods (i.e., divergent primer PCR with outward facing primers as well as RNase R exonuclease treatment for the enrichment of circular RNAs) while elucidating the functions of HBV RNA-interacting proteins. Importantly, these circular RNAs could be detected not only in the cell culture model that mimics HBV infection and in the recently developed minicircular HBV model that mimics HBV cccDNA persistent infection but also in human primary hepatocytes naturally infected with HBV *in vitro*, suggesting the possibility that these RNA forms are produced in HBV-infected human liver tissues.

In general, several mechanisms for the production of exonic circular RNAs have been proposed, such as back-splicing or exon skipping [16]. Regardless of the mechanism, short repetitive sequences are believed to be frequently involved in circular RNA production [19]. Although HBV pgRNA is intron-less, pgRNA contains conserved regions of 11-mer pair direct repeat sequences at the 5’ and 3’ ends, designated DR1 and DR2. Additionally, based on the terminal redundancy in pgRNA, the 60-mer stem-loop structure (ɛ element), which also contains a series of inverted repeats, is present at both ends of pgRNA. Furthermore, the DHX9 protein, which was found to interact with HBV RNA in this study, was recently reported to bind to inverted repeat elements, and loss of the DHX9 protein led to an increase in the number of circular RNAs [13], which is consistent with the present results showing that DHX9 knockdown resulted in increased levels of HBV circular RNA. Although the mechanism underlying how HBV circular RNA is produced remains to be elucidated, the repeat sequences of both the HBV pgRNA and the RNA binding protein DHX9 may be involved in the production of viral circular RNA in HBV infection.

The functions of circular RNAs are generally unknown. However, some circular RNAs may work as miRNA sponges, regulators of transcription, or as templates for protein translation [16]. Because we previously found that some sequences in the HBV RNA may work as decoys for specific miRNAs [4], it may be interesting to determine whether the circular RNA identified here also functions as a miRNA sponge and, consequently, involving in the hepatocarcinogenesis. As for regulators of transcription, it is generally thought that when mRNAs are transformed into circular RNAs, they act as an “mRNA trap” by sequestering the translational start site [20]. Although this may be involved in the decreased levels of viral protein synthesis shown in this study, further examination is needed to clarify whether knockdown of DHX9 protein levels leads to decreased levels of viral protein and/or HBV mRNA levels by directly regulating the production of viral circular RNA. In terms of templates for translation, some circular RNAs were recently reported to be associated with translating ribosomes [21]. In fact, we also found by RIP assay that the ribosomal protein P0/P1/P2 might be associated with viral circular RNAs. However, this does not always mean that the RNA is actually translated. With the exception of a few specific circular RNAs [21], most circular RNAs are generally considered “translational silent” [16], and this may be true in this case as well, because we found that the increased production of viral circular RNA by DHX9 knockdown led to decreased levels of viral proteins. Identification of the levels of viral circular RNA bound to polysomes may be necessary to further clarify whether viral circular RNA is translated.

Clinically, even though similar HBV-DNA levels are detected in the sera, HBsAg levels differ significantly across cases, and the reason for this is unknown. Although it was recently reported that the use of RNA interference-based therapy against HBV transcripts with integrated HBV DNA is the source of HBsAg [22], the differences in the viral circular RNA levels or DHX9 protein levels may also be involved in these differences (Supplementary Figure 4). Although this idea is interesting, quantification of the absolute value of the small number of molecules will be critical. The ddPCR method applied here in primary hepatocytes and the HBV infection model may be useful for future accurate analyses of clinical samples.

Whereas we focused on DHX9 in this study, several other proteins were also found to associate with HBV RNAs. Examination of these proteins in the context of HBV infection also requires further study. Nonetheless, we found that RNAs important in the HBV life cycle and pathogenesis are promising targets for the development of novel therapeutics against HBV.

## MATERIALS AND METHODS

### Cells

HepAD38 cells, which express HBV pgRNA under the control of the inducible tetracycline promoter, were kindly provided by Prof. C. Seeger [12]. The cells were maintained in Ham's F-10/DMEM culture media supplemented with 10% doxycycline-free fetal bovine serum (FBS) with or without 1 μg/ml doxycycline to maintain the repression of HBV expression or to maintain HBV replication, respectively. HepG2 cells were purchased from the American Type Culture Collection (Manassas, VA, USA) and were cultured in Dulbecco's Modified Eagle's Medium (DMEM) media supplemented with 10% FBS. Primary human hepatocytes isolated from chimeric uPA/SCID mice with humanized livers using the collagenase perfusion method [23, 24] were obtained from Phoenix Bio (Hiroshima, Japan). The purity of human hepatocytes was greater than 95%. A total of 3.0 × 10^5^ cells/well were seeded on a type I collagen-coated 24-well plate and maintained in dHCGM (DMEM with 10% FBS, 5 ng/ml epidermal growth factor, 0.25 μg/ml insulin, 0.1 mM ascorbic acid, and 2% dimethyl sulfoxide) [25]. These cells were able to support the long-term replication of HBV infection *in vitro*. All cells were incubated at 37° C, 20% O_2_, and 5% CO_2_.

### Plasmids

The TL7 plasmid, which expresses HBV pgRNA (genotype D: GenBank accession number; V01460), was kindly provided by Prof. Loeb [26]. Minicircle HBV cccDNA with a Gaussia luciferase reporter (mcHBV-GLuc cccDNA)-containing plasmid (pre-mcHBV-GLuc) was kindly provided by Prof. Su [27]. This plasmid harbors the sequences of HBV genotype C. HBV pgRNA-expressing plasmid (genotype C) was kindly provided by Prof. Mizokami [28].

### HBV minicircle extraction

Minicircle mcHBV-GLuc cccDNA was produced using the Minicircle DNA Vector Technology MC-Easy System (System Biosciences, Mountain View, CA, USA) from ZYCY10P3S2T *Escherichia coli* transformed with pre-mcHBV-GLuc, which expresses inducible ƟC31 integrase and I-SceI endonuclease to eliminate the bacterial plasmid DNA backbone. No contamination of genomic and parental DNA was confirmed by agarose gel electrophoresis. The Gluc signal comes from pgRNA, providing a surrogate of cccDNA activity, and it secretes from the cells into the media. Gluc activity in the aliquots of the media was measured using the reagent for measurement of renilla luciferase activity in the Dual Reporter Assay system (Promega, Madison, WI, USA) because *Gaussia* and *Renilla* luciferases catalyze the light reaction using the same substrate.

### Transfection

HepG2 cells were transfected with mcHBV-GLuc- or HBV pgRNA-expressing plasmids using Effectene transfection reagent (Qiagen, Hilden, Germany) according to the manufacturer's instructions.

### *In vitro* transcription and 3′ end biotinylation

To perform RNA pull-down, we produced HBV-RNAs containing S, X, or core regions by an *in vitro* transcription reaction using MEGAScript (Ambion, Austin, TX, USA) according to the manufacturer's instruction. Briefly, PCR amplification of the corresponding regions using the TL7 plasmid as a template was performed with T7 promoter sequences in the forward primers. The primers used were: S region, Fw, 5′-TAATACGACTCACTATAGGGAGAATGGGGCAGAATCTTTCCACC-3′ and Rv, 5′-TTAAATGTATACCCAAAGACAAAAG-3′, X region, Fw, 5′-TAATACGACTCACTATAGGGAGAATGGCTGCTAGGCTGTGCTG-3′ and Rv, 5′-TTAGGCAGAGGTGAAAAAGTTG-3′, and Core region, Fw, 5′-TAATACGACTCACTATAGGGAGAATGGACATCGACCCTTATAAAGA-3′ and Rv, 5′-CTAACATTGAGATTCCCGAGATT-3′. PCR products were purified by gel extraction using the DNA MinElute kit (Qiagen), followed by *in vitro* transcription. *In vitro* transcribed RNAs were treated with DNase, purified using MEGAclear Transcription Cleanup Kit (Ambion), and subjected to ethanol precipitation. Aliquots were run in a denatured gel to confirm the integrity of the obtained RNA. Biotinylated cytidine bisphosphate was attached to the 3′ end of the 50 pmol RNAs by T4 RNA ligase using Pierce RNA 3′ End Biotinylation Kit (Thermo Fisher, Waltham, MA, USA).

### RNA pull-down and mass spectrometry

To screen proteins interacting with RNAs, the Pierce Magnetic RNA-Protein Pull-Down Kit was used (Thermo Fisher) according to the manufacturer's instructions. HepAD38 cell lysates without HBV expression were prepared by adding M-PER Mammalian Protein Extraction Reagent (Thermo Fisher; 300 μl for 10^7^ cells). Streptavidin beads and biotinylated RNAs containing HBV S, X, and core regions were mixed, followed by the addition of 60 μl of cell lysate to the each mixture for 60 min at 4° C. After five washes, 25 μl of elution buffer was added and subjected to SDS-PAGE. After gel electrophoresis, proteins were stained with the Silver Staining Kit for Mass Spectrometry (Wako, Osaka, Japan). Possible specific bands, determined by comparing the results from the negative control (sample without RNA), were excised and the proteins were identified by liquid chromatography tandem mass spectrometry (LC-MS/MS; APRO Life Science Institute, Tokushima, Japan). The resulting tryptic peptides were separated and analyzed using reversed phase capillary high-performance liquid chromatography directly coupled to a Finnigan LCQ ion trap mass spectrometer (LC-MS/MS) with a slight modification. The individual MS/MS spectra were processed using TurboSEQUEST software (Thermo Quest, San Jose, CA, USA). The generated peak list files were used to query either the MSDB database or NCBI using the MASCOT program (http://www.matrixscience.com).

### Western blot analysis and antibodies

Western blotting was performed as previously described [29]. To confirm the proteins identified by LC-MS/MS, RNA immunoprecipitation samples were transferred to polyvinylidene fluoride (PVDF) membranes (GE Healthcare, Chalfont St. Giles, UK) after SDS-PAGE. In other cases, lysate samples were separated by 10–20% gradient SDS-PAGE followed by electrical transfer to PVDF membranes. After blocking with 5% dry milk, membranes were probed with the appropriate primary antibodies diluted in Immunoshot Reagent 1 (Cosmo Bio, Tokyo, Japan) overnight at 4° C. Horseradish peroxidase (HRP)-conjugated corresponding secondary antibodies (GE Healthcare) were subsequently used. Trueblot anti-rabbit IgG HRP (1:1000; Rockland, Limerick, PA, USA) was used as the secondary antibody to avoid interfering with the immunoprecipitated immunoglobulin heavy and light chains. Bound antibodies were detected using Immunostar LD reagents (Wako). The following antibodies were used: HBV PreS2 (#ab30923, 1:1000), DNA-PK (#ab32566, 1:1000), DHX9 (#ab26271, 1:1000), ILF3 (#ab92355, 1:1000), and snRNP200 (#ab118713, 1:1000) from Abcam (Cambridge, UK); leucine-rich PPR motif-containing protein (LRPPRC; #ARP41093, 1:1000) from AVIVA Systems Biology (San Diego, CA, USA); and β-actin (#5125, 1:2000) from Cell Signaling Technology (Danvers, MA, USA).

### RNA immunoprecipitation

To examine the endogenous binding of candidate proteins to HBV RNAs, the RIP assay kit (MBL, Nagoya, Japan) was used according to the manufacturer's instructions. Briefly, 6.0 × 10^6^ HepAD38 cells with HBV expression were seeded on 10-cm dishes. Cells were harvested and lysed, followed by the addition of precleared Protein G agarose beads for 1 h. The supernatants were mixed with antibodies against candidate proteins or control rabbit IgG and agarose beads for 4 h at 4° C. The pellets were washed four times, and bound RNA was isolated by ethanol precipitation. To detect bound RNAs, 1 μg of precipitated RNA and 5% input were reverse-transcribed to cDNA using SuperScript III Reverse Transcriptase (Invitrogen, Carlsbad, CA, USA), and semi-quantitative RT-PCR was performed. The primers used were as follows: S, Fw, 5′-ACCTCTATGTATCCCTCCTG-3′ and Rv, 5′-GACTCAAGATGCTGTACAGAC-3′; X, Fw, 5′-ACTCTCTCGTCCCCTTCTCCG-3′ and Rv, 5′-AGGCAGAGGTGAAAAAAGTTGC-3′; and core, Fw, 5′-ACAGTTATAGAGTATTTGGTG-3′ and Rv, 5′-GAGATCTTCTGCGACGCGGCG-3′. The antibodies used were: snRNP200 (A303-454A) and LRPPRC (A304-731A) from Bethyl Laboratories (Montgomery, TX, USA), DNA-PK (#ab70250) from Abcam, and DHX9 (RN063PW) from MBL.

For the RIP assay, RNase R treatment, and RT-PCR, 1 × 10^7^ HepAD38 cells were harvested and subjected to the RIP assay according to the manufacturer's protocol. The antibodies used were anti-DHX9 (RN063PW) and anti-ribosomal protein P0/P2/P3 antibodies (RN004M, MBL), which were RIP-certified. After immunoprecipitation, samples were divided and subjected to western blotting for confirmation of immunoprecipitation or subjected to RNA extraction. Three micrograms of RNA were either reverse transcribed or treated with RNase R, purified, and reverse transcribed. PCR was performed using the indicated primers.

### Gene knockdown

shRNA-expressing lentiviruses against each protein were purchased from Santa Cruz Biotechnology (Dallas, TX, USA): shDNA-PK (sc-35200-V), shsnRNP200 (sc-75243-V), shILF3 (sc-106301-V), shDHX9 (sc-45706-V), and shLRPPRC (sc-44734-V). HepAD38 cells were transduced with these viruses using polybrene, followed by puromycin selection to establish stably shRNA-expressing cells. In the case of primary hepatocytes, coinfection with HBV was done without selection.

### Hirt extraction and Southern blotting

HepAD38 cells were harvested, and HBV DNA, including cccDNA, was extracted with the Hirt method [30] for Southern blotting to extract only viral-derived DNAs. Briefly, SDS is applied to break down the lipid membranes and viral capsids to release all viral nucleic acids. A high concentration of salt is then added to precipitate high-molecular-weight cellular chromatin and protein covalently bound to DNA in SDS–protein complexes. HBV cccDNA, a protein-free DNA, is one of the major viral products in the supernatant, which can be purified by organic phenol. Another protein-free viral DNA, relaxed circular DNA (rcDNA), which is a precursor of cccDNA, can be extracted simultaneously.

Southern blotting was performed using digoxigenin (DIG)-labeled probes (Roche Diagnostics, Basel, Switzerland) according to the manufacturer's instructions. Briefly, DNA samples were separated on an agarose gel. After depurinating the DNA in 250 mM HCl and denaturing in 0.5 M NaOH/1.5 M NaCl, DNA was transferred to a Hybond N+ membrane (GE Healthcare) with the capillary transfer method in 20× SSC. After UV crosslinking, the membrane was incubated with a DIG-labeled DNA probe at 42° C overnight. After hybridization, membranes were stringently washed at room temperature in 2× SSC containing 0.1% SDS and at 65° C in 0.5× SSC containing 0.1% SDS, twice each, and the bound probe was visualized with a DIG wash and block buffer set (Roche Diagnostics), according to the manufacturer's instructions. The DNA marker was purchased from Roche Diagnostics (DIG-labeled DNA Molecular Weight Marker VII).

To synthesize an RNA probe for Southern blotting, HBV full-length RNA was synthesized by *in vitro* transcription and a DIG RNA labeling kit (Roche Diagnostics). The template was a PCR product with T7 primer. The primers used were: Fw, 5′-TAATACGACTCACTATAGGGGGTGCGCAGACCAATTTATGC-3′ and Rv, 5′-GCACCATGCAACTTTTTCAC-3′.

### RT-PCR and RNase R treatment

Reverse transcription was performed using SuperScript III First-Strand Synthesis Supermix (Invitrogen). Oligo d(T) or random primers were used for priming as described in the figure legends. In the case of RNase R treatment, RNA was incubated with RNase R (Epicentre, Madison, WI, USA), which specifically digests linear RNAs, at 10 U/μg for 15 min [15], followed by purification using the RNeasy MinElute Cleanup kit (Qiagen). After purification, reverse transcription using random primers was performed to generate cDNAs.

PCR was performed using the primers described in Supplementary Figure 3. Primers for the host gene Mcl1 were used as a control for linear mRNA. The sequences were as follows: Fw, 5′-GTGCAGCGCAACCACGAGAC-3′ and Rv, 5′-GCAGCACATTTCTGATGCCG-3′. RNA without reverse transcription was used as the template for negative controls.

### Northern blotting

Northern blotting was performed as described previously [29], with slight modification. Briefly, 5 μg of RNAs with or without RNase R treatment was separated in a 1% formaldehyde denatured agarose gel. Before transfer, the gel was stained briefly with ethidium bromide to confirm RNA integrity. The RNAs were hydrostatically transferred to a Hybond N+ membrane (GE Healthcare), and the membranes were UV-crosslinked and prehybridized in hybridization buffer. Hybridization was performed overnight at 42° C in ULTRAhyb Buffer (Ambion) containing 10 ng/ml DIG-labeled RNA probes, which had been denatured at 90° C for 10 min. Each membranes were stringently washed twice at 55° C in 2× SSC containing 0.1% SDS and in 0.1× SSC containing 0.1% SDS, and the bound probe was visualized using a DIG wash and block buffer set (Roche Diagnostics) according to the manufacturer's instructions. The RNA probes, from position 1901 to position 2701, corresponding to the core region of the pgRNA, were synthesized by *in vitro* transcription using MEGA Script (Ambion) following PCR amplification of the region with T7 promoter-containing primers. The primers were as follows: Fw, 5′-ATGGACATCGACCCTTATAAA-3′ and Rv, 5′-TAATACGACTCACTATAGGGTAAGGTTTAATACCCTTATCC-3′.

### *In vitro* HBV infection

Human primary hepatocytes were infected with five equivalent copies of HBV (genotype C) (PhoenixBio) with 40% polyethylene glycol 8,000 (Hampton Research, Aliso Viejo, CA, USA), as described previously [31]. dHCGM media (PhoenixBio) were changed every 5 days. Seventeen days after infection, cells were harvested and subjected to RNA extraction for RT-PCR or protein extraction for western blotting.

### TaqMan real-time PCR and ddPCR

For TaqMan RT-PCR, 1.0 μl of template was subjected to quantitative real-time PCR using SYBR universal mix 2 (Applied Biosystems, Foster City, CA, USA) with the StepOnePlus real-time PCR system. The threshold cycle number (Ct), at which the amount of amplified target reached a fixed threshold, was measured. Real-time PCR experiments were performed in triplicate for each sample.

ddPCR was performed using the QX100 Droplet Digital PCR system (Bio-Rad Laboratories, Hercules, CA, USA). Briefly, 1.0 μl of template cDNA reverse transcribed from 3 μg of RNA treated with DNase and RNase R with 20× primer and a TaqMan probe set was partitioned into approximately 20,000 droplets by the QX100 Droplet Generator (Bio-Rad Laboratories). Cycling conditions were as follows: 95° C for 10 min, followed by 45 cycles of 94° C for 30 s and 56° C for 1.5 min, and a final 10 min incubation at 98° C. The droplets were subsequently read automatically by the QX10 droplet reader. Assays were performed in duplicate for each sample. The data were analyzed with QuantaSoft analysis software 1.3.2.0 (Bio-Rad Laboratories). Duplicate data were combined into a single metawell before Poisson analysis. The probe set was designed to amplify between positions 1,561 and 1,883 of the HBV genome (Fw, 5′-CTTCTCATCTGCCGGACC-3′ and Rv, 5′-CACAGCTTGGAGGCTTGA-3′), and the detection probe was FAM-5′-AGGCTGTAGGCATAAATTGGTCT-3′-BHQ, which covers the HBV-DNA gap, as described previously [32].

### Statistical analysis

Statistically significant differences between groups were identified using Student's *t*-test when the variances were equal. When the variances were unequal, Welch's *t*-test was used instead. *P* values less than 0.05 were considered to indicate statistical significance.

## SUPPLEMENTARY MATERIALS FIGURES



## Abbreviations

HBV: Hepatitis B virus
cccDNA: Covalently closed circular DNA
DXH9: DExH-box helicase 9
pgRNA: Pregenomic RNA
miRNAs: MicroRNAs
shRNA: Short hairpin RNA
DNA-PK: DNA-dependent protein kinase
snRNP200: Small nuclear ribonucleoprotein 200 kDa
ILF3: Interleukin enhancer binding factor 3
RT-PCR: Reverse transcription-PCR
RIP: RNA-immunoprecipitation
ddPCR: Droplet-digital PCR
FBS: Fetal bovine serum
PVDF: Polyvinylidene fluoride.
