# Supplementary material for: DHX9 regulates production of hepatitis B virus-derived circular RNA and viral protein levels
Source: Oncotarget. 2018 Apr 20;9(30):20953–64. doi: 10.18632/oncotarget.25104 (PMC5940377; doi:10.18632/oncotarget.25104)
Supplement: Supplementary file 1 [file oncotarget-09-20953-s001.pdf]

DHX9 regulates production of hepatitis B virus-derived circular RNA and viral protein levels

SUPPLEMENTARY MATERIALS

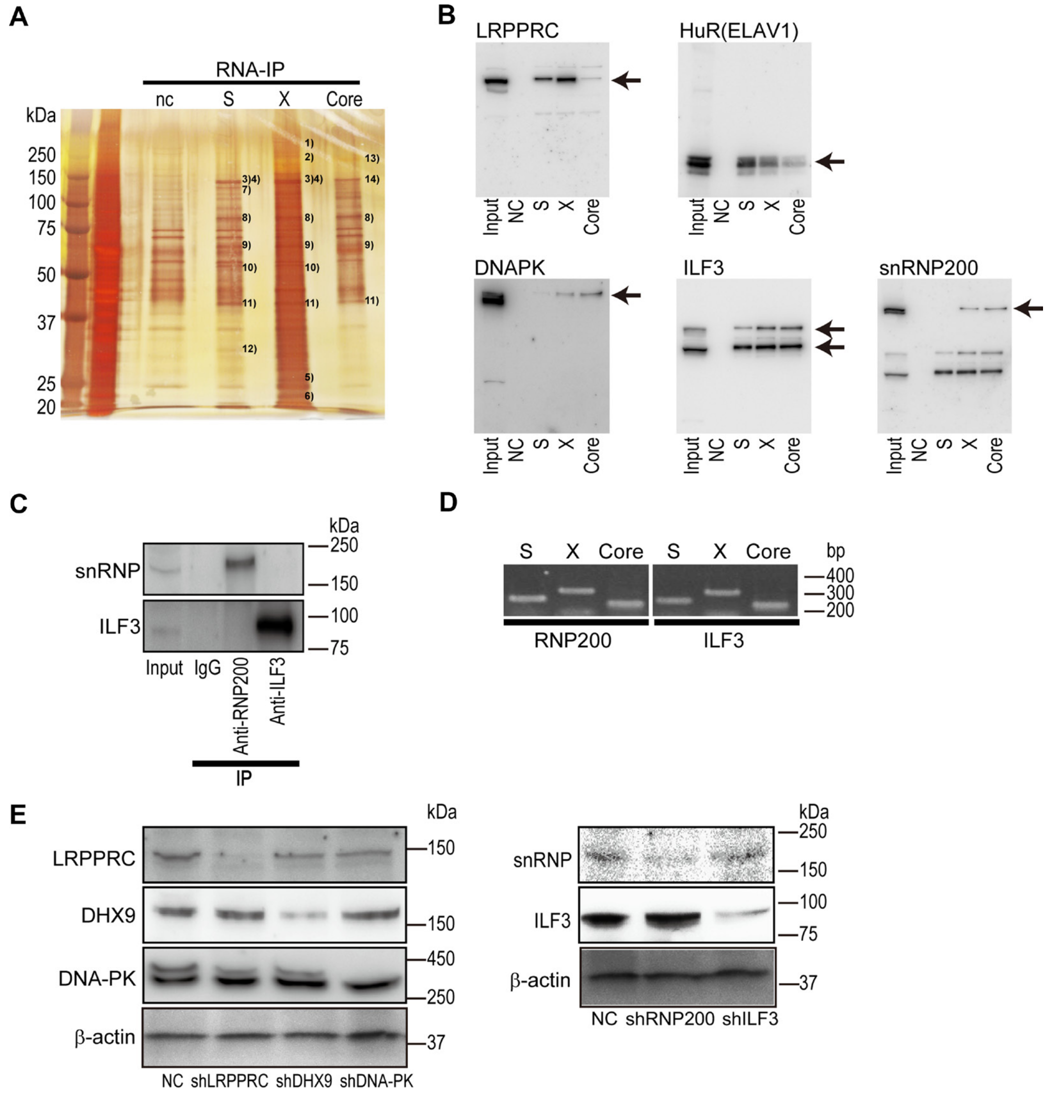

Supplementary Figure 1: HBV RNAs are associated with host proteins. (**A**) Silver staining image after *in vitro* RNA immunoprecipitation for mass spectrometry. Biotinylated HBV RNAs corresponding to the S, X, or Core regions were mixed with HepG2 cell lysates and precipitated, followed by sodium dodecyl sulfate-polyacrylamide gel electrophoresis. The bands with numbers were considered possible candidates and excised for mass analyses. Bands with the same numbers were considered the same and mixed for identification. nc, negative control (no RNA). (**B**) Western blotting to confirm the results of the mass analyses. Samples used for silver staining were blotted with antibodies against the indicated proteins. Five percent of the cell lysates was used as an input. (**C**) HepAD38 cells with HBV expression were used for the immunoprecipitation of the indicated proteins. Western blotting was used to confirm the immunoprecipitation of the targeted proteins. Five percent of the cell lysates was used as an input. Normal rabbit IgG was used as a negative control. A representative image of two independent experiments is shown. (**D**) RNA was extracted from the immunoprecipitations by the indicated proteins and was subjected to RT-PCR to determine if the indicated RNA regions (S, X, or Core) were included. A representative image of two independent experiments is shown. (**E**) HepAD38 cells were transduced with the indicated shRNA-expressing lentiviruses and selected. Knockdown of each protein indicated was confirmed by western blotting. A representative image of two independent experiments is shown.

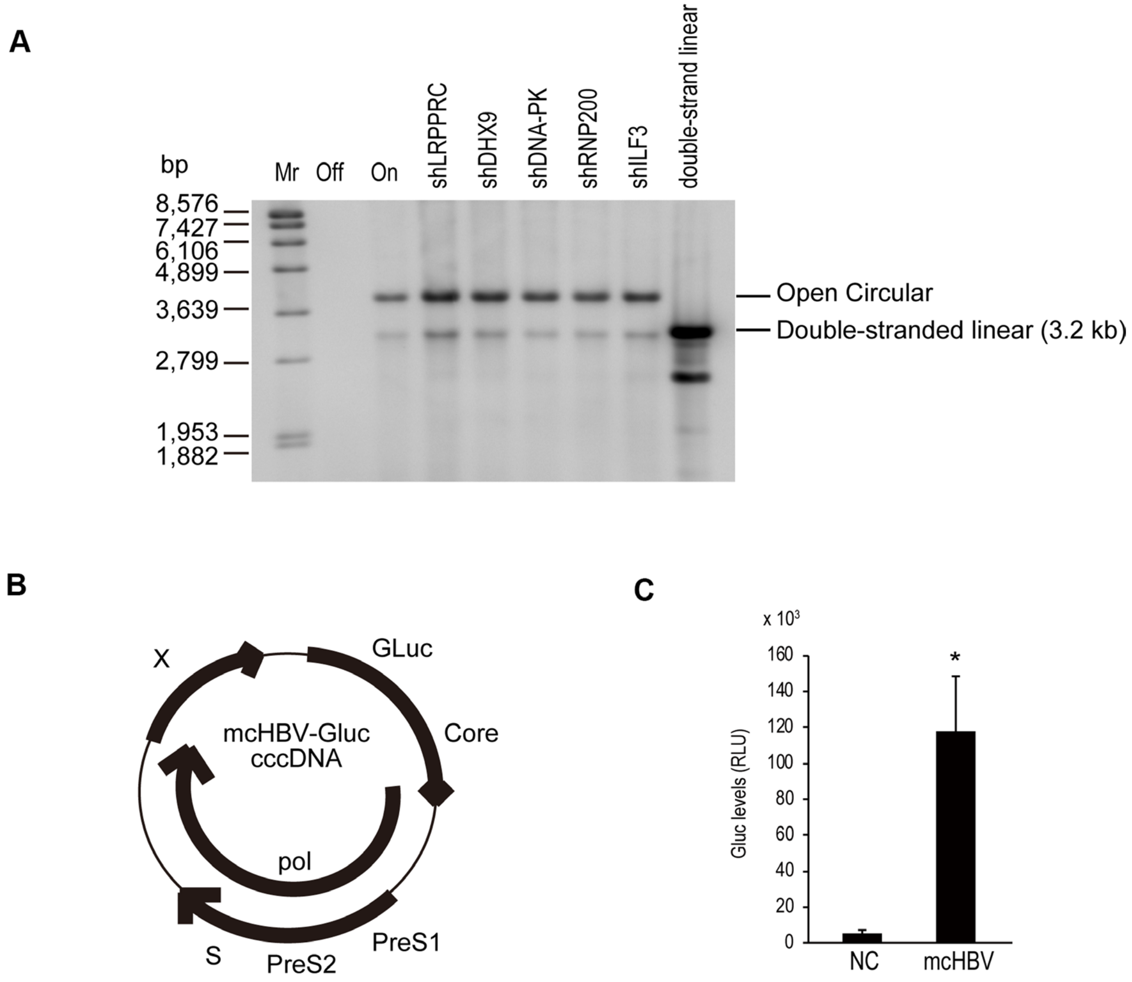

Supplementary Figure 2: HBV-DNA levels were not significantly affected by knockdown of the identified RNA binding proteins. (**A**) HepAD38 cells with the indicated knockdown construct were used for HBV-DNA Southern blotting. HBV expression was shut off for 2 days by adding 1 μg/ml doxycycline, followed by DNA extraction and Southern blotting. HepAD38 cells without HBV expression (Off) and with constitutive HBV expression (On) were used as controls. A double-stranded linear marker was also applied. The upper panel is the image after a short exposure, and the lower panel is that after a longer exposure. The position of cccDNA is estimated from the pattern of the obtained bands and the size marker. A representative image of two independent experiments is shown. (**B**) The HBV minicircle construct used is depicted. (**C**) HepG2 cells were transiently transfected with the HBV minicircle construct, and Gluc luciferase activity was measured after 3 days. Data represent the mean ± SE of two independent experiments. $^{*}p < 0.05$.

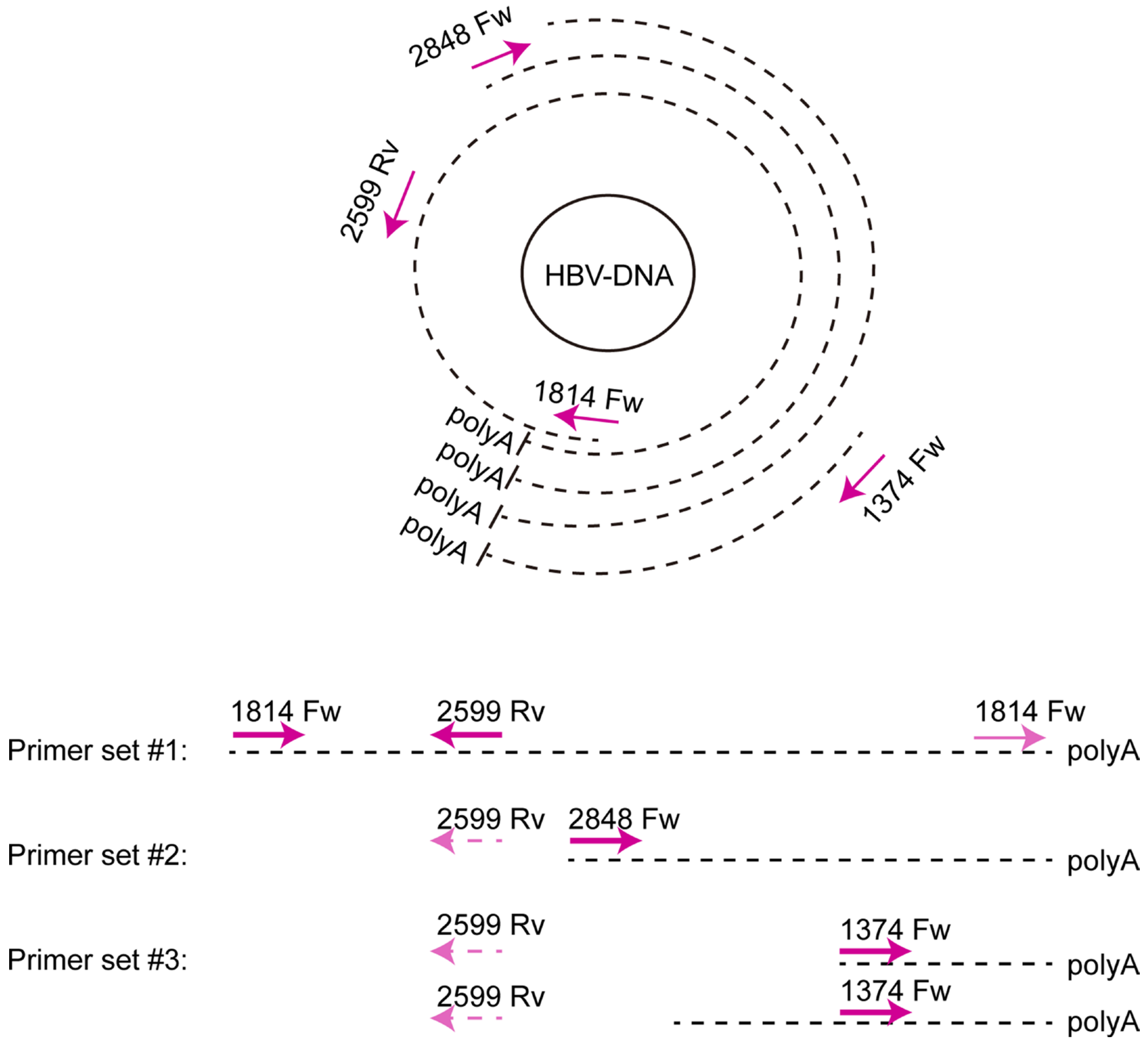

Supplementary Figure 3: Positions of the primers used in this study. Primer positions used in this study are indicated by arrows with numbers. Four kinds of HBV mRNAs (precore mRNA is not drawn) are indicated by dotted lines. Fw, forward, Rv, reverse. Primer set #1 used the primers 1814 Fw and 2599 Rv. Primer set #2 used the primers 2848 Fw and 2599 Rv. Primer set #3 used the primers 1374 Fw and 2599 Rv. Lower figures indicate the primer positions of the three primer sets on the linear HBV mRNAs.

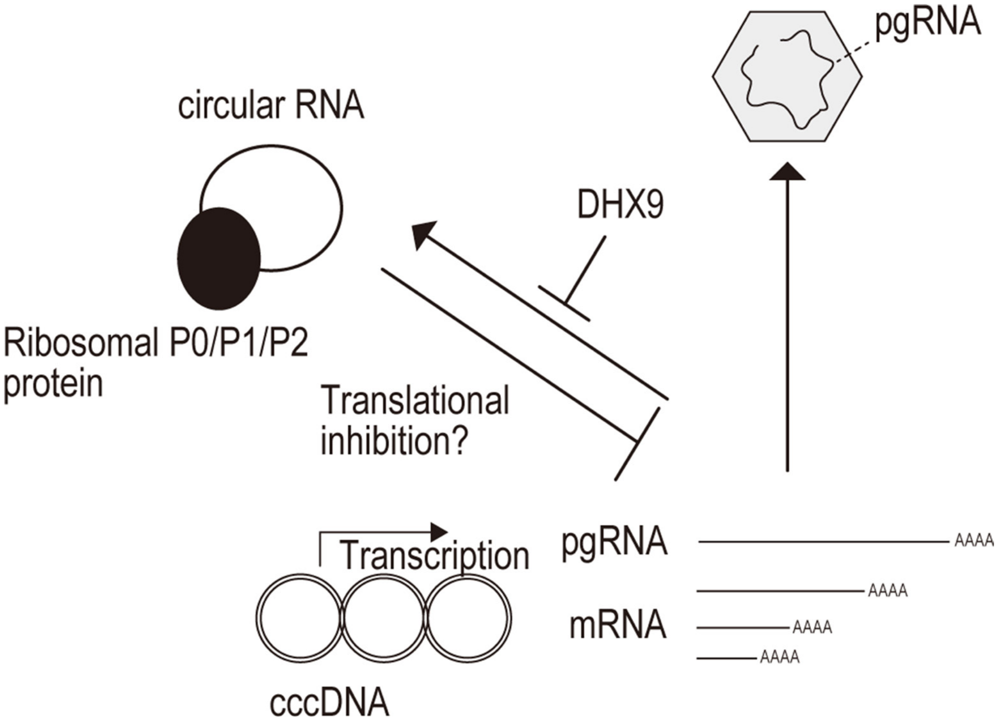

Supplementary Figure 4: Proposed model in this study. Viral-derived circular RNA is produced during HBV replication, and the production is inhibited by the DHX9 protein. Circular RNA binds with ribosomal protein, which may work as a decoy for the ribosomal protein, resulting in the inhibition of viral proteins. The differences in the viral circular RNA levels or DHX9 protein levels may be involved in the differences of the viral protein expression levels.
